# The differential effect of chronological age and brain age on cognitive fatigue: new metrics, new insights

**DOI:** 10.1007/s00415-025-13584-1

**Published:** 2026-01-07

**Authors:** G. R. Wylie, C. A. F. Román, J. C. Buckey, N. D. Chiaravalloti, M. J. Falvo, J. C. Ford, H. M. Genova, C. E. Niemczak, R. M. Roth, J. DeLuca

**Affiliations:** 1 Kessler Foundation, West Orange, NJ USA; 2 Rutgers University New Jersey Medical School, Newark, NJ USA; 3 Department of Veterans Affairs, War Related Illness and Injury Study Center, NJ Health Care System, East Orange, NJ USA; 4 Geisel School of Medicine at Dartmouth, 1 Medical Center Drive, Lebanon, NH USA; 5 Dartmouth-Hitchcock Medical Center, Lebanon, NH USA; 6 Keck School of Medicine of the University of Southern California, Los Angeles, CA USA; 7 Dartmouth College Department of Psychological and Brain Sciences, Hanover, NH USA

**Keywords:** Cognitive Fatigue, Brain Age, Aging, MRI

## Abstract

Fatigue is prevalent in the general population, but it is unclear whether aging is associated with increased fatigue. Here, we investigate the relationship between cognitive fatigue (CF, fatigue resulting from mental work) and two types of aging—chronological age and brain age—in 85 participants ranging in age from 20 to 84 years. Whereas chronological age is simply participants’ absolute age, brain age is derived from a comparison of participants’ brain morphology relative to a normative model. CF was induced using a working memory paradigm that participants repeatedly performed, reporting their instantaneous level of CF at baseline and after each successive block of the task. Chronological age was associated with a decrease in the CF reported at baseline (the intercept of a regression line fit to the CF ratings), whereas brain age was related to the rate at which fatigue was induced (the slope of the regression line fit to the CF ratings). Behaviorally, the decrease in CF as a function of chronological age was mirrored by a more liberal response bias, providing more evidence that response bias represents an objective behavioral index of CF. Additionally, areas of the insula showed a relationship between CF and chronological age, suggesting that the role of the insula may change across the lifespan. These results represent the first well-powered study to investigate the relationship between CF and chronological age as well as brain age and suggests that CF may be an important indicator of brain age across the lifespan.

**Supplementary Information:**

The online version contains supplementary material available at 10.1007/s00415-025-13584-1.

## Introduction

While fatigue is a significant problem for individuals with central nervous system (CNS) injury or disease [1–4], it is also prevalent in the general population [5, 6] with as many as 21.9% of the population reporting high levels of trait fatigue (i.e., persistent fatigue, present on most days) [7]. There is widespread belief that older individuals report more fatigue than their younger counterparts, which may be due to a combination of physiological, neuromuscular, and systemic changes, such as age-related hormonal changes [8, 9]. One such study found that approximately 40% of individuals aged 65–92 years reported high levels of general fatigue [10]. Others have shown that the elderly report less fatigue than younger participants and that fatigue decreases across time [11]. Cross-sectional studies have shown an inverse correlation between fatigue and age [12, 13], which supports the idea that elderly individuals report less fatigue than younger individuals. One potential explanation of this counterintuitive finding, as well as for the mixed findings in the literature, is that it is not simply chronological age that is related to fatigue, but also “brain age”—or the apparent age of one’s brain, which may be older or younger than one’s chronological age [e.g., 14, 15]. According to this hypothesis, the expectation of a positive correlation between age and fatigue derives from an intuition that individuals who show signs of accelerated aging (that is, a relative difference in age rather than an absolute difference) should experience more fatigue than those who do not. One of the primary aims of this paper is to investigate the relationship between chronological age and a widely used estimate of brain age.

We have introduced several metrics to better understand cognitive fatigue (CF, a psychobiological state caused by prolonged cognitive activity), which all rely on the use of a fatigue induction paradigm: participants repeatedly perform a moderately challenging working-memory task and report the intensity of CF across intervals over time. Since participants are asked to report their current level of CF, this paradigm measures *state* CF, as opposed to *trait* measures of CF, which are assessed with retrospective self-report measures that are more susceptible to self-report biases. Using a fatigue induction paradigm, we have shown a set of brain areas where activation changes as a function of CF known as the “fatigue network”: the caudate nucleus of the basal ganglia, the ventro-medial prefrontal cortex (vmPFC), the dorsolateral prefrontal cortex (DLPFC), the anterior cingulate cortex (ACC), and the insula [see also 15–17]. We have also shown that by fitting a regression line to each participant’s CF values, the slope (CF.slope) and intercept (CF.intercept) represent useful summary measures of state CF [18].

In an effort to identify objective behavioral metrics of CF, we have also shown that the metrics of signal detection theory (SDT) covary with CF [19, 20]. Signal detection theory provides a framework to analyze participants’ errors, allowing inferences to be made about participants’ response bias and perceptual sensitivity [21]. Response bias can be liberal (participants correctly respond to many target stimuli, but also make false alarms by responding to non-targets) or conservative (participants miss some target stimuli, but also make few false alarms). Another SDT metric is perceptual sensitivity, or participants’ ability to distinguish targets from non-targets. Our work showed that as participants reported more CF, they adopted a more conservative response ‘criterion’ (i.e., bias). Additionally, increasing CF was associated with decreasing perceptual sensitivity, replicating previous findings [e.g., 22].

Here, we investigate the effects of both chronological age and brain age on CF using the expanded set of analytic tools we have developed. We hypothesize that CF will show an inverse correlation with chronological age, as in our previous work [13]. To investigate brain age, we used Gaussian process regression via brainageR to obtain a predicted brain age from each participant’s high-resolution anatomical scan (i.e., 3D T1-MPRAGE) based on a model trained on 3377 “healthy” individuals [23–25]. This allowed us to compute a brain-predicted age difference (Brain-PAD) score for each participant: the difference between their brain age, as predicted by the brainageR pipeline and their chronological age. We hypothesize a positive relationship between Brain-PAD scores and CF. For both chronological age and Brain-PAD, correlations were calculated for both CF.slope and CF.intercept to understand which aspect of CF was relevant. We further hypothesize that age will show a relationship with CF-related activation in the fatigue network. Additionally, because we have found that lower CF is related to a more liberal response bias (using SDT metrics), we hypothesize that older individuals will adopt a more liberal response bias than the young since they report less CF.

## Materials and methods

### Participants

This is a secondary analysis of six datasets, including 85 participants, ranging in age from 20 to 84 years of age (see Table 1), who were originally recruited as “healthy controls”. Accordingly, all participants were free of all psychiatric diagnoses (e.g., schizophrenia, bipolar disorder, major depressive disorder) and neurological disease (e.g., multiple sclerosis) or injury (e.g., traumatic brain injury, stroke). They were right-handed, were free of drug or alcohol abuse history, and not taking any medication that would affect cognition or the hemodynamic response (e.g., benzodiazepines, neuroleptics, or psychostimulants), and free of MRI contraindications. All participants signed an informed consent form approved by the IRB at the relevant institution (see Supplementary data section, Table S1, for the institution where each of the six studies was undertaken), and all were paid for their participation.

**Table 1:** Demographics of the sample

	Female (*N* = 46)	Male (*N* = 39)	Overall (*N* = 85)
Age			
Mean (SD)	51.0 (18.2)	42.2 (12.0)	46.9 (16.2)
Median [min, max]	54.0 [22.0, 84.0]	42.0 [20.0, 71.0]	43.0 [20.0, 84.0]
Education			
Mean (SD)	16.3 (2.36)	15.3 (2.26)	15.9 (2.35)
Median [min, max]	16.0 [12.0, 22.0]	16.0 [11.0, 21.0]	16.0 [11.0, 22.0]

The age, education, and sex of the participants is shown. Age and education are in years

*SD* standard deviation

The number of participants and tasks performed in each study are reported in Table 2.

**Table 2:** The number of participants who performed each of the tasks and number of task blocks in each of the studies

Study	Number of participants	Tasks performed	Number of task blocks
Study 1	30	0-back, 2-back	4, 4
Study 2	15	0-back, 2-back	4, 4
Study 3	14	0-back, 2-back	4, 4
Study 4	7	0-back, 2-back	4, 4
Study 5	9	2-back	6
Study 6	10	2-back	4
Total	85		

“0-back” and “2-back” were conditions of the *N*-back working memory paradigm

## Experimental design

### 2-Back and 0-back tasks

Two conditions of the *N*-back working memory task were used: the 0-back and 2-back conditions. In both conditions, a series of 65 letters were presented in white on a black computer screen, one after another. Each letter was presented for 1.5 s, followed by an inter-trial interval of 500 ms during which a fixation cross was presented. The 0-back and 2-back conditions differed, in that, for the easier 0-back task, participants were asked to respond each time the target letter “K” was presented. For the more difficult 2-back task, participants were asked to respond when the target letter was the same as the letter presented two trials before in the sequence (e.g., N, Q, R, Q…). In both conditions, 16 of the 65 stimuli were targets. The time between successive trials in both conditions was jittered using the Optseq2 program to optimize later deconvolution of the fMRI data (https://surfer.nmr.mgh.harvard.edu/optseq/). Each block lasted 4 min, 30 s. Participants reported their instantaneous level of CF at baseline and after each block. All procedures and paradigms were standardized across sites.

### CF assessment

To assess state CF, participants were asked to rate their perceived level of CF using a visual-analog scale of fatigue (VAS-F). All participants were instructed to rate the CF they were experiencing “right now, at this moment”. For studies 2–5, the ratings were recorded using a two-button response box: the button on the left moved a cursor to the left and the button on the right moved the cursor to the right along a horizontal line presented at the center of the screen. The position of the cursor was converted to a percentage of the length of the horizontal line, and this was used as participants’ CF rating. For study 1, the ratings were made verbally: the experimenter asked the participant to rate his/her CF as a number between 0 (no CF) and 100 (maximal CF). This was accompanied by written instructions above a horizontal line with 0 at the extreme left end and 100 at the extreme right.

To assess trait CF, participants completed either the Modified Fatigue Impact Scale (MFIS; studies 1–5) [26] or the Chalder Fatigue Scale (CFS; study 6) [27]. Both scales assess trait fatigue by asking participants to rate different aspects of their fatigue over the past 4 weeks, but studies suggest that they assess different aspects of trait fatigue [e.g., 28]. Therefore, only the MFIS was used for the analysis of trait CF. In all cases, these questionnaires were completed after the MRI scan.

### Magnetic resonance imaging acquisition

MRI data were obtained using 3.0 T scanners. In all cases, participants were instructed to minimize head motion and foam padding was used for additional movement restriction. For all studies, high-resolution anatomical images were acquired a using magnetization-prepared rapid gradient-echo sequence (MP-RAGE). The parameters used for each study are shown in the Supplementary Data section, Table S2. Functional imaging was performed using gradient echo, T2*-weighted echo-planar (EPI) images. The parameters used for each study were optimized for each site and, as Table S2 shows (see Supplementary Data), were comparable across sites.

## Statistical analyses

Statistical analyses were conducted using the R statistical package (version 4.3.0). Prior to analysis, the normality of all variables was assessed by visual inspection and the Agostino test [29]. In those cases when the data were found to be skewed, they were transformed using the Box–Cox method [30].

### State CF (VAS-F scores)

For the analysis of the VAS-F scores, a linear mixed effects analysis (LME) was used. The intensity of CF experienced during each block of each task was estimated by calculating the average of the VAS-F score recorded before and after each block, and this was used as the dependent measure. The factors were Task (0-back vs. 2-back), Block (block 1–4+), and Session (Session 1 vs. 2, see below). Age was included as a quantitative variable; participant and site (KF, UMDNJ, DHMC) were included as independent, random factors. The factor Session was included in the model because in some studies participants performed the same tasks on separate occasions (sessions). The median time between sessions was 29 days (range = 14–73 days).

### CF.slope and CF.intercept

The slope and intercept of CF was derived by fitting a regression line to the VAS-F scores for each task and participant [3, 18]. The extent to which the slope and intercept differed across participants was investigated using an LME with the factors of Task and Session (as above). Age was included as a quantitative variable; participant and site were independent, random factors. For the analysis of CF slope, the intercept was also included in the model to account for the fact that high initial CF values (the intercept) constrain the extent to which CF can increase during CF induction.

### Trait CF

Most of the participants in the study completed the MFIS (*n* = 72), while a subset completed the CFS (*n* = 10). To assess the effect of age on trait CF, the data from the cognitive subscale of the MFIS were analyzed with a linear model in which the score was predicted by Age. In addition, we tested for a relationship between state and trait CF measures by adding CF.intercept to the same model. This analysis was done for completeness and is reported in the supplementary data section.

### Response time (RT) and accuracy

Response time and accuracy were analyzed with an LME that included the factors of Task, Block, and Session (as above); Age and the VAS-F scores were included as quantitative variables; participant and site were included as independent, random factors. For RT, only RTs from correct trials were included in the analysis. Accuracy was calculated as the proportion of trials on which the correct response was given in each block relative to the total number of trials in that block. These analyses were done for completeness and are reported in the supplementary data section.

### SDT measures (*d*′ and criterion)

Using signal detection theory, discrimination certainty and response bias were calculated. These two factors can independently affect accuracy [31, 32]. The SDT calculations relied on the false-alarm rate (FA; the proportion of responses made to stimuli that were not targets), the hit rate (HR; the proportion of correct identifications of target stimuli), and the inverse of the standard normal cumulative distribution (*z*). The discriminability index (*d*′), or the ability to correctly identify target stimuli, was calculated as [*z*HR − *z*FA]. Because all stimuli were high contrast and clearly identifiable, *d*′ is best thought of as perceptual certainty. Response bias was measured using ‘criterion’, calculated as − 1/2[*z*HR + *z*FA]. Higher values of criterion represent a more conservative response bias, because participants made fewer false alarms but also registered fewer hits. Lower values represent a more liberal response bias because participants registered more hits, but also made more false alarms.

For each of the SDT measures (perceptual certainty [*d*′] and response bias [Criterion]), an LME was used with the factors of Task, Block, and Session (as above). Age and VAS-F were quantitative variables; participant and site were included as independent, random factors.

### Brain-predicted age difference (Brain-PAD)

Each participant’s brain age was estimated using brainageR (v2.1) [23–25], which is a voxel-based morphometry derived model available pre-trained by a machine learning pipeline from a large multi-age set of training images (for more information, see https://github.com/james-cole/brainageR (10.5281/zenodo.3476365). The model predicted each participant’s brain age based on their T1-MPRAGE, with a 95% confidence interval. The brain-predicted age difference (Brain-PAD) was then calculated by subtracting each participant’s chronological age from their predicted brain age. This resulted in difference scores such that positive values reflected an “older” brain and negative values reflected a “younger” brain.

The resulting Brain-PAD scores were used for two analyses, after being checked for outliers using a Rosner test (number of suspected outliers = 13, corresponding to the number of Brain-PAD scores ≥  ± 10 years). The Rosner test showed none of the data to be outliers. We then investigated whether Brain-PAD predicted CF.slope and CF.intercept in separate models, including chronological age as a covariate.

### Neuroimaging processing and analysis

The neuroimaging data were preprocessed using the fMRIPrep processing pipeline (version 1.4.1; Esteban et al. [33]). A full description of the preprocessing steps can be found in the supplemental material (see Text S1) and included slice timing correction, spatial realignment, co-registration to the T1 MPRAGE image, nonlinear warping into standard MNI space, estimation of confounds such as framewise displacement (FD), DVARS [34], and global signals associated with white matter, cerebrospinal fluid, and a mask of the whole brain. A set of physiological regressors was also extracted to allow for component-based noise correction (*CompCor* [35]). The data were smoothed to minimize anatomical differences and increase the signal to noise ratio (6 mm FWHM); then each voxel was scaled to the grand mean intensity of that voxel across the acquisition run. A deformation field to correct for susceptibility distortions was estimated based on field maps that were co-registered to the BOLD reference images using a custom workflow of fMRIprep [36].

Prior to deconvolution, the motion data were analyzed to compute the Euclidian distance between each successive acquisition in the BOLD time series. Any acquisitions in which the Euclidian distance was more than 1.0 mm were excluded (censored) from the deconvolution. Blocks in which 25% or more of the acquisitions were censored were considered excessively contaminated by motion and excluded from the group-level analysis. This criterion resulted in the exclusion of 9.5% of the data. In the deconvolution, motion parameters and their derivatives from the realignment step were used as regressors of no interest, as were FD and the first six components from aCompCor. Each block of the 0- and 2-back tasks were modeled separately. Because the analyses of CF.intercept and CF.slope showed that only CF.intercept was affected by age, we focused on CF.intercept in the analysis of the neuroimaging data. Accordingly, the beta weights from the correct trials of only the first block of the neuroimaging data were used in the group-level analyses.

As in previous research, we modeled the data with an LME using the 3dLMEr script in the AFNI suite of processing tools. In this analysis, the factors were Task (0-back, 2-back) and Session (session 1 vs. 2). The quantitative variables were Age and CF.intercept. Participant and site were modeled as random factors.

The results were corrected for multiple comparisons by using an individual voxel probability threshold of *p* < 0.005 and a cluster threshold of 25 voxels (voxel dimension = 3.4 × 3.4 × 4 mm). Monte Carlo simulations, using 3dClustSim (version AFNI_24.0.17, compile date: Mar 24, 2024) showed this combination resulted in a corrected whole brain alpha level of *p* < 0.05. Because we had prior hypotheses about the areas in the fatigue network, we also calculated the cluster threshold for this set of regions of interest (ROIs). These calculations showed that a cluster threshold of ten voxels resulted in a corrected alpha level of *p* < 0.05 for areas within the fatigue network.

## Results

### Demographics

A *t*-test showed that the women in our sample were significantly older than the men (*t*(78.6) = 2.68, *p* = 0.009, *η*^2^ = 0.08). This is shown in Table 1: women had a mean age of 51.0 years, while men had a mean age of 42.2 years. This raised the possibility that in any analysis in which age had a significant effect, the older cohort would be more representative of women and the younger cohort would be more representative of men. All analyses were therefore run both with and without sex as a covariate. In no case did the inclusion of sex change the pattern of results. We report the results of the analyses without sex as a covariate here. There was no significant relationship between educational attainment and age.

### State CF (VAS-F)

**Fig. 1:**
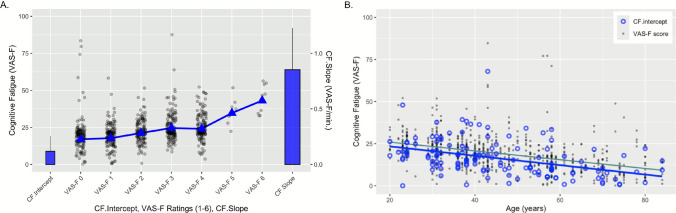
CF ratings as a function of run (left) and as a function of age (right). Panel **A** shows subjects’ ratings across the runs of the experiment (gray points). The mean of the each rating is indicated by the blue triangle. Also shown are the mean CF.intercept (far left) and mean CF.slope (far right). The CF.intercept is on the same scale as the VAS-F ratings; the CF.slope is plotted against the scale on the right edge of panel **A**. The error bars represent the 95% confidence interval. Panel **B** shows the relationship between CF and age. The VAS-F ratings are shown in gray points, as is the best fitting regression line. Also shown is the relationship between CF.intercept and age. The CF.intercepts are plotted in open blue circles and the best fitting regression line is shown in blue

There was a main effect of Block (*F*(6,676.2) = 4.81, *p* < 0.0001, *η*^2^ = 0.04), resulting from increasing CF across the blocks (see Fig. 1A). There was also a main effect of Age (*F*(1,104.1) = 6.98, *p* = 0.01, *η*^2^ = 0.06), resulting from a negative relationship between CF and age (coefficient = − 0.50): for every year of increased age, participants reported 0.50 less CF (see Fig. 1B). No other effects or interactions were significant.

### CF.intercept and CF.slope

For CF.intercept, the effect of Age was significant (*F*(1,81.9) = 4.96, *p* = 0.03, *η*^2^ = 0.06; see Fig. 1B). This was due to a negative relationship between Age and CF.intercept (coefficient = − 0.33), showing that participants’ “baseline” CF decreased with age. No other effects or interactions were significant.

For CF.slope, the only significant effect was Session (*F*(1,115.7) = 7.15, *p* = 0.009, *η*^2^ = 0.06). The CF.slope was higher for session 2 (0.021) than for session 1 (0.006). No other effects or interactions were significant.

### Brain-PAD

**Fig. 2:**
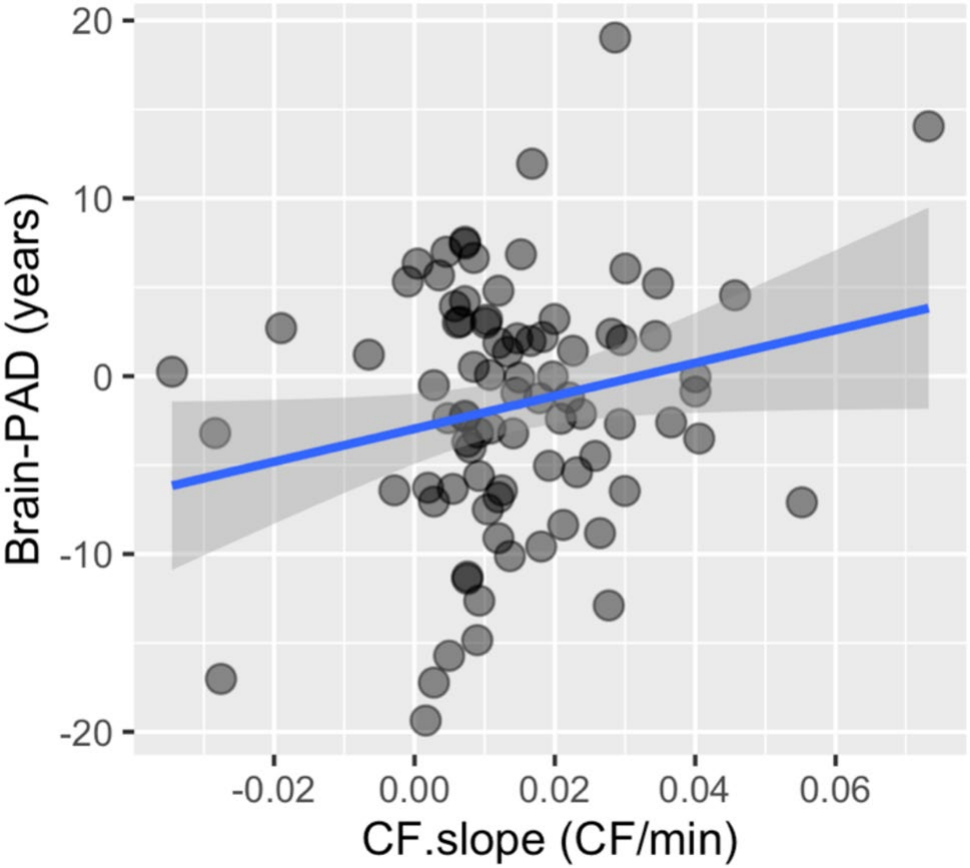
The relationship between Brain-PAD (in years) and CF.slope (in CF change per minute). The blue line represents the best-fitting regression line and the shaded area around the line represents the 95% confidence interval

The linear model predicting CF.slope showed a significant effect of Brain-PAD (*F*(1,82) = 3.98, *p* = 0.05, *η*^2^ = 0.05), which was due to a positive relationship between CF.slope and Brain-PAD (coefficient = 0.0005). That is, participants with brains that were “younger” than their chronological age fatigued at a slower rate than participants with “older” brains. This can be seen in Fig. 2. To estimate the predictive accuracy of our model relative to models with other combinations of the variables in our model, we used *k*-fold cross-validation. We cross-validated the full model and models with every possible combination of the variables in the full model using leave-one-out cross-validation. The model with the lowest mean squared error (MSE) was the model in which CF.slope was predicted by Brain-PAD alone and the associated coefficient was 0.0005. The variance (standard deviation) in the MSEs of the models was 0.000004.

Unlike for CF.slope, the linear model predicting CF.intercept showed no effect of Brain-PAD.

### Signal detection theory (SDT) metrics

**Fig. 3:**
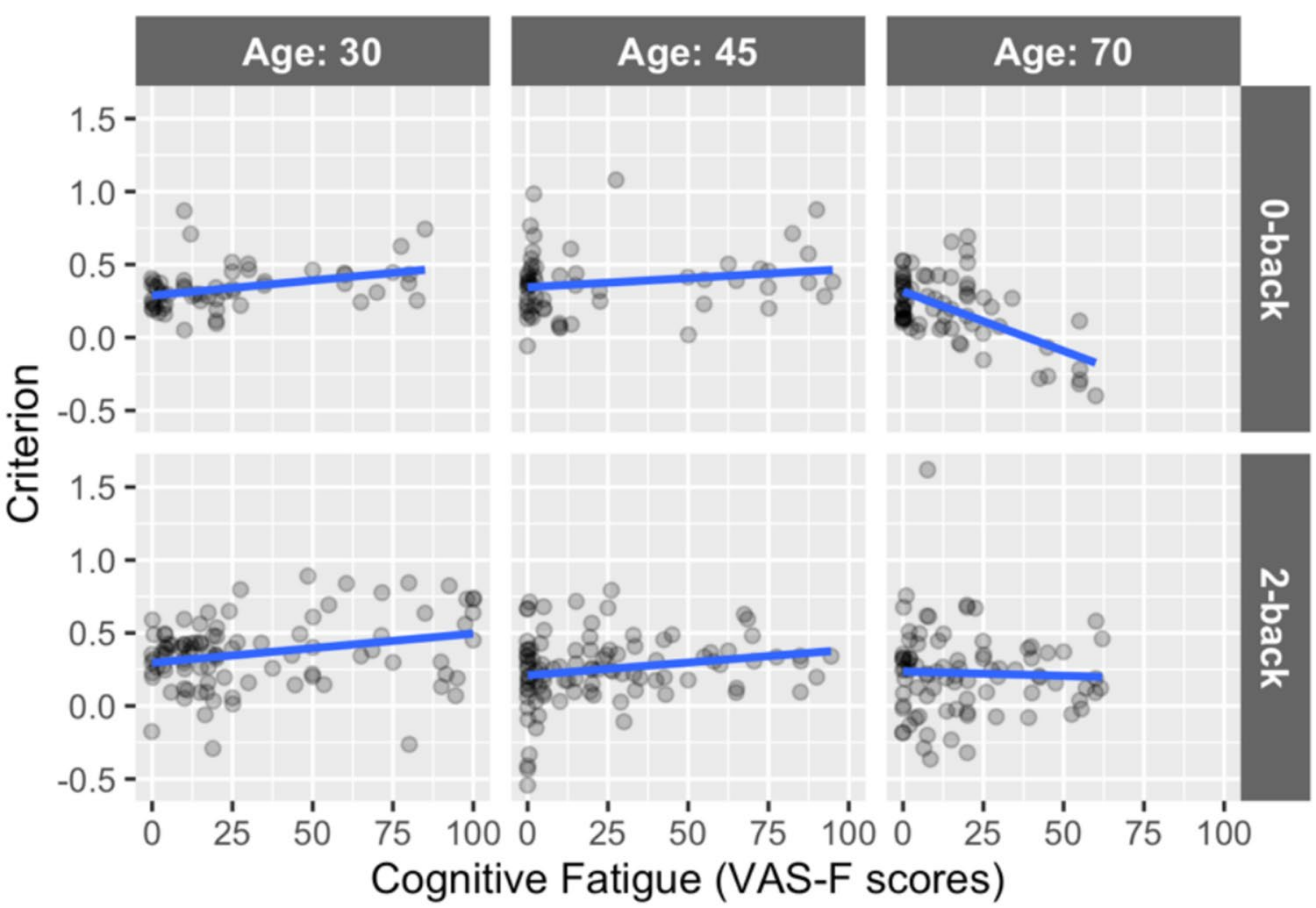
The between CF, age, and task in the analysis of criterion. The continuous variable age has been divided into tertiles for exposition only (ages 20–39, 40–70, and 71+), with each tertile represented by a column: “Age: 30” shows ages 20–39, “Age: 45” shows ages 40–65, “Age: 70” shows ages 66+. The blue lines show the best-fitting regression line for each subset of the data (again, for exposition only). The 0-back task is shown in the top row and the 2-back task is shown in the bottom row

Analysis of the Criterion data showed an effect of CF (*F*(1,287.1) = 5.03, *p* = 0.03, *η*^2^ = 0.02) resulting from a positive relationship between the VAS-F scores and Criterion (coefficient = 0.0008)—that is, as CF increased, participants adopted a more conservative response strategy, replicating previous findings [20]. Additionally, there was an interaction between CF and Age (*F*(1,335.8) = 4.56, *p* = 0.03, *η*^2^ = 0.01) and a three-way interaction between CF, Age, and Task (*F*(1,402.3) = 6.10, *p* = 0.01, *η*^2^ = 0.01). As Fig. 3 shows, this three-way interaction resulted from a positive relationship between Criterion and CF when participants were young (as CF increased, participants became more conservative) that became increasingly flat as age increased; for the 0-back task, the slope became negative for older individuals (as CF increased, participants became more liberal). Additionally, the effect of Session (*F*(1,63.4) = 9.89, *p* = 0.003, *η*^2^ = 0.14) was also significant and resulted from participants responding with a more conservative response bias on session 1 (*c* = 0.44) than on session 2 (*c* = − 0.04). No other effects or interactions were significant.

For perceptual certainty (*d*′), there were main effects of CF (*F*(1,281.4) = 5.36, *p* = 0.02, *η*^2^ = 0.02), Task (*F*(1,397.3) = 12.59, *p* = 0.0004, *η*^2^ = 0.03), and Session (*F*(1,62.9) = 8.72, *p* = 0.004, *η*^2^ = 0.12). The effect of CF resulted from a negative relationship between d’ and CF scores (coefficient = − 0.004), meaning that the more CF participants reported, the worse was their perceptual certainty. The effect of Task resulted from participants showing higher certainty on the 0-back task (*d*′ = 4.27) than on the 2-back task (*d*′ = 3.18). The effect of Session resulted from participants responding with more certainty in session 2 (*d*′ = 4.21) than in session 1 (*d*′ = 3.24). No other effects or interactions were significant.

### Neuroimaging results

**Fig. 4:**
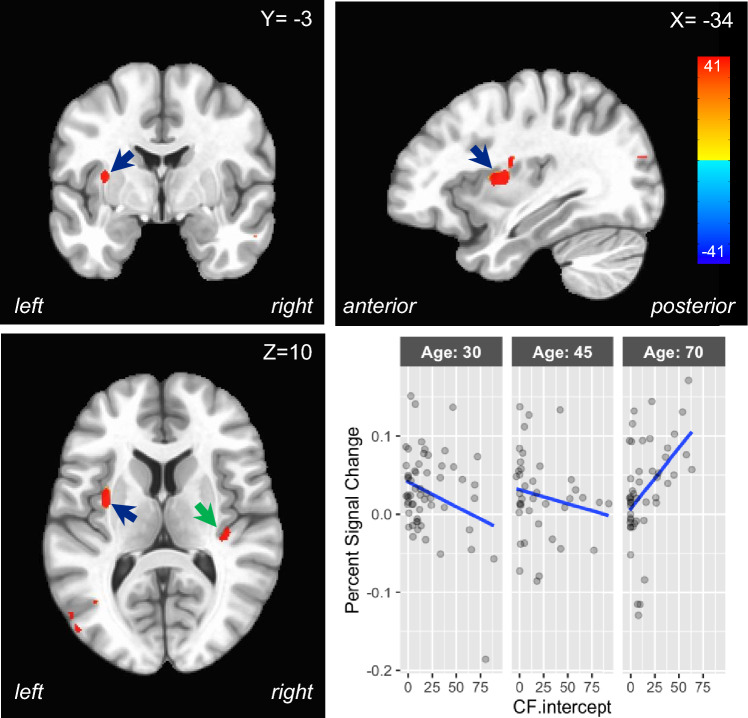
Brain areas showing a significant interaction between CF.intercept and age in the BOLD activation. Three orthogonal views are shown (*X* *Y* *Z* = − 34, − 3, 10). The interaction between CF.intercept and age is shown in the left insula (blue arrow) and the right insula (green arrow). The inset graph shows the relationship between CF.intercept and percent signal change in the left insula for younger (ages close to 30), middle-aged (ages close to 45) and older (ages close to 70) individuals. The division of ages into three plots is for exposition only: in the analysis, age was a continuous variable

In the neuroimaging results, there was an interaction between Age and CF.intercept (see Table 3 and Fig. 4). Three of the areas associated with CF.intercept were part of the fatigue network: the ACC and the insula bilaterally. Moreover, as Fig. 4 shows, the relationship between CF.intercept and percent signal change changed as a function of age: younger individuals showed a negative relationship, while older individuals showed a positive relationship. As Table 3 shows, this was true for all three regions.

**Table 3:** Brain areas showing a significant interaction between CF.intercept and age in the BOLD activation

						Age tertile		
Location	*X*	*Y*	*Z*	Voxels	*χ*^2^ stat	30	45	70
Middle/anterior cingulate cortex	− 13.5	12.4	42	14	16.149	− 0.0009	0.0001	0.0019
Insula lobe	− 34.1	− 4.8	10	13	21.765	− 0.0012	− 0.0001	0.0018
Rolandic operculum/insula lobe	41.5	− 25.4	18	22	17.139	− 0.0018	− 0.0002	0.0024
Parahippocampal gyrus	− 16.9	− 39.2	− 6	39	20.614	− 0.0023	− 0.0003	0.0030
Inferior temporal gyrus	38.1	− 56.4	− 2	27	22.787	− 0.0016	0.0000	0.0026
Middle occipital gyrus	− 41.0	− 87.3	22	31	40.710	− 0.0021	0.0004	0.0048

*X* *Y* *Z* = the location of the voxel with peak intensity in each cluster; *N* voxels refers to the number of voxels in the cluster; *χ*^2^ stat refers to the maximal *χ*^2^ statistic in each cluster. The age tertiles show the coefficient of the best-fitting regression line in each of three age segments. N.B., this is for expository purposes only; in the analysis, age was entered as a continuous variable

## Discussion

Our results replicate previous investigations of the effects of age on state CF by showing a negative relationship between age and fatigue (see Fig. 1B) [11–13]. Furthermore, our results show that the critical difference between younger and older individuals is that chronologically older individuals have lower levels of CF when they start performing the task (as Fig. 1B shows, the CF.intercept was negatively correlated with age). To our knowledge, this is the first time this effect has been reported. Furthermore, the analyses of the Brain-PAD data showed that the “older” the brain age, rather than chronological age, the more quickly did CF accrue: the first time this has been shown to our knowledge. The relationship between Brain-PAD and CF.slope suggests that CF.slope may represent a proxy or marker for brain age and overall brain health. To better understand how much variance in Brian-PAD was explained by CF.slope, we calculated the correlation of these two variables (based on the finding that the model that included only these two variables had the lowest MSE). The correlation was 0.21, which means that the amount of variance in Brain-PAD explained by CF.slope was a fairly modest ~ 5%. Thus, CF.slope could be one important, low-cost tool to help assess brain health in the future. Taken together, these results suggest that baseline CF rates decrease with age, and that CF accrues at a faster rate in individuals with accelerated brain aging. This suggests that there are some cases in which brain age is more informative than chronological age (e.g., CF.slope) and that two individuals with the same chronological age but with different Brain-PAD scores may well experience different rates of CF accrual.

Accuracy data, acquired during performance of a working memory task, showed a relationship with CF that seems intuitive: higher levels of CF were associated with lower accuracy (see supplementary data). However, this relationship was affected by age in a surprising way—for older individuals, increased CF was associated with increased accuracy (see Figure S1). The SDT analyses may help to explain this counterintuitive finding. As we have shown in previous work [19, 20], CF was positively related to Criterion, meaning that as participants became more fatigued, they adopted a more conservative response strategy. However, this was true only for the younger participants. For older participants, there was little relationship between CF and Criterion for the more difficult 2-back task, while for the easier 0-back task the relationship was markedly negative (see Fig. 3). That is, as older participants became more fatigued, they adopted a more liberal response strategy, particularly for the 0-back task. Given the low difficulty of the 0-back task, this was a good strategy because it did not result in increased errors (in fact, the reverse was the case). The perceptual certainty data (*d*′) replicated previous results, showing that as CF increased, *d*′ decreased [20, 22]. More broadly, these results support the idea that SDT metrics represent objective behavioral indices of CF and support our hypothesis that because older individuals report less CF, they should also exhibit a more liberal response bias.

We found no evidence to support the idea that trait CF was affected by age (see supplemental data). The discrepancy between findings based on state measures relative to trait measures underscores the sensitivity and advantage of state measures of CF. We also found no evidence for a relationship between state and trait measures of CF in this dataset, replicating previous findings [37].

The neuroimaging findings confirmed our hypothesis that areas within the fatigue network would be sensitive to age-related changes in CF. Specifically, the insula/putamen and the ACC showed an interaction between age and CF such that more activation was associated with less fatigue in the younger participants, but more activation was associated with more fatigue in the older participants (see Fig. 4). The interpretation of this pattern of results must remain speculative, but it is possible that the role of these areas changes across the lifespan. In the young, activation in these areas seems to combat CF, while in older adults, activation in these areas seems to monitor CF. If this interpretation is correct, one implication is that attempting to combat CF by activating these areas results in more CF than using these areas to monitor CF. This may explain why mindfulness techniques have shown some efficacy in decreasing fatigue [38].

Although this study resulted in several new insights regarding CF, it also had some limitations. For example, the sample was not large enough to draw conclusions about the prevalence of CF in the elderly population, and relationships shown at the population level (such as the extent to which CF.slope predicts Brain-PAD) do not necessarily translate into clinically actionable inferences at the individual level. Furthermore, the demographic information about our sample did not include factors such as race or socioeconomic status, which potentially limits generalizability. Moreover, we also lacked participant anthropometrics (i.e., body mass index) which has been shown at a population level to influence brain age [39]. 

## Conclusions

This study shows, for the first time, that the rate of CF increase (CF.slope) is related to Brain-PAD, suggesting that CF.slope may represent a biomarker for brain health. We also replicate previous work showing that older individuals report less CF than their younger counterparts and extend this work in several important ways. We show that the decrease in CF in older participants is due to lower baseline CF (CF.intercept). We also show that the decrease in CF as a function of age is mirrored by a more liberal response bias, providing more evidence that the metrics of SDT provide objective behavioral indices of CF. Finally, this study sheds some light on the mechanism of the decrease in CF with increasing age, suggesting that less CF is reported when the insula and ACC monitor CF than when these brain areas are used to combat CF. The finding that CF accrues at different rates in individuals with similar chronological ages, but different Brain-PAD scores, is important for CF research, since it helps to explain some of the variance in CF scores which may have led to the disparate findings in the literature. Furthermore, this finding could also be important clinically, since a steeper CF.slope may indicate worse brain health than a shallower CF.slope regardless of chronological age.

## Supplementary Information

Below is the link to the electronic supplementary material.

Supplementary file1 (DOCX 348 KB)

## Data Availability

The data used in this study are available on request. Open data sharing is not possible, given the sensitive nature of participants’ data and the language in the informed consent that each participant signed. However, deidentified derivative data will be made available by the lead author (GRW) upon formal request indicating name and affiliation of the researcher as well as a brief description of the intended use for the data. All requests will be required to comply with Kessler Foundation procedures.
