# Supplementary material for: The differential effect of chronological age and brain age on cognitive fatigue: new metrics, new insights
Source: J Neurol. 2026 Jan 7;273(1):63. doi: 10.1007/s00415-025-13584-1 (PMC12779680; doi:10.1007/s00415-025-13584-1)
Supplement: Supplementary file 1 — Supplementary file1 (DOCX 348 KB) [file 415_2025_13584_MOESM1_ESM.docx]

Table S1. The tasks performed and the intuitions where each study was conducted.

| **Study** | **Tasks performed** | **Institution(s)** | **VAS-F acquisition** |
| --- | --- | --- | --- |
| Study1 | 0back, 2back | Kessler Foundation and Rutgers University | Verbal |
| Study2 | 0back, 2back | Kessler Foundation | Button-box |
| Study3 | 0back, 2back | Kessler Foundation | Button-box |
| Study4 | 0back, 2back | Kessler Foundation | Button-box |
| Study5 | 2back | Kessler Foundation | Button-box |
| Study6 | 2back | Dartmouth College | Button-box |

Table S2. The acquisition parameters of the neuroimaging data for each study

| **Study** | **Scanner** | **Anatomical parameters** | **Functional parameters** |
| --- | --- | --- | --- |
| Study1 | Siemens Allegra | TR=2000 ms, TE=4.38 ms, flip angle=8°, effective TI=900 ms, matrix=256x256, FOV=220 mm, 144 slices, slice thickness=1 mm, in-plane resolution=1x1 mm | TR=2000 ms, TE=30 ms, flip angle=80°, matrix=64x64, FOV=22 cm, 32 slices, slice thickness=4 mm, in-plane resolution=3.438x3.438 mm |
| Study2 | Siemens Skyra | TR=2100 ms, TE=3.43 ms, flip angle=9°, effective TI=900 ms, matrix=256x256, FOV=256 mm, 176 slices, slice thickness=1 mm, in-plane resolution=1x1 mm | TR=2000 ms, TE=30 ms, flip angle=90°, matrix=92x92, FOV=22 cm, 32 slices, slice thickness=3.99 mm, in-plane resolution=2.391x2.391 mm |
| Study3 | Siemens Skyra | TR=2100 ms, TE=3.43 ms, flip angle=9°, effective TI=900 ms, matrix=256x256, FOV=256 mm, 176 slices, slice thickness=1 mm, in-plane resolution=1x1 mm | TR=1500 ms, TE=30 ms, flip angle=90°, matrix=96x96, FOV=19.2 cm, 44 slices, slice thickness=2.5 mm, in-plane resolution=2x2 mm |
| Study4 | Siemens Skyra | TR=2100 ms, TE=3.43 ms, flip angle=9°, effective TI=900 ms, matrix=256x256, FOV=256 mm, 176 slices, slice thickness=1 mm, in-plane resolution=1x1 mm | TR=1500 ms, TE=30 ms, flip angle=90°, matrix=96x96, FOV=19.2 cm, 44 slices, slice thickness=2.5 mm, in-plane resolution=2x2 mm |
| Study5 | Siemens Skyra | TR=2100 ms, TE=3.43 ms, flip angle=9°, effective TI=900 ms, matrix=256x256, FOV=256 mm, 176 slices, slice thickness=1 mm, in-plane resolution=1x1 mm | TR=1500 ms, TE=30 ms, flip angle=70°, matrix=96x96, FOV=19.2 cm, 44 slices, slice thickness=2.5 mm, in-plane resolution=2x2 mm |
| Study6 | Siemens Prisma FIT | TR=2530 ms, TE=3.63 ms, flip angle=7°, effective TI=1110 ms, matrix=256x256, FOV=256 mm, 176 slices, slice thickness=1 mm, in-plane resolution=1x1 mm | TR=1500 ms, TE=30 ms, flip angle=65°, matrix=80x80, FOV=24 cm, 56 slices, slice thickness=3 mm, in-plane resolution=3x3 mm |

**Text S1. Neuroimaging preprocessing steps**

For anatomical preprocessing, the T1-weighted (T1w) image from each subject was corrected for intensity non-uniformity (INU) with N4BiasFieldCorrection [12], distributed with ANTs 2.2.0 [1, RRID:SCR_004757], and used as T1w-reference throughout the workflow. The T1w-reference was then skull-stripped with a *Nipype* implementation of the antsBrainExtraction.sh workflow (from ANTs), using OASIS30ANTs as target template. Brain tissue segmentation of cerebrospinal fluid (CSF), white-matter (WM) and gray-matter (GM) was performed on the brain-extracted T1w using fast (FSL 5.0.9, RRID:SCR_002823, [13]). Volume-based spatial normalization to one standard space (MNI152NLin2009cAsym) was performed through nonlinear registration with antsRegistration (ANTs 2.2.0), using brain-extracted versions of both T1w reference and the T1w template. The following template was selected for spatial normalization: *ICBM 152 Nonlinear Asymmetrical template version 2009c* [[4], RRID:SCR_008796; TemplateFlow ID: MNI152NLin2009cAsym].

The following preprocessing was performed for each of the BOLD runs for each participant for each task block. First, a reference volume and its skull-stripped version were generated using a custom methodology of *fMRIPrep*. The BOLD reference was then co-registered to the T1w reference using flirt (FSL 5.0.9, [7]) with the boundary-based registration [5] cost-function. Co-registration was configured with nine degrees of freedom to account for distortions remaining in the BOLD reference. Head-motion parameters with respect to the BOLD reference (transformation matrices, and six corresponding rotation and translation parameters) are estimated before any spatiotemporal filtering using mcflirt (FSL 5.0.9, [6]). BOLD runs were slice-time corrected using 3dTshift from AFNI 20160207 ([3], RRID:SCR_005927). The BOLD time-series (including slice-timing correction when applied) were resampled onto their original, native space by applying a single, composite transform to correct for head-motion and susceptibility distortions. These resampled BOLD time-series will be referred to as *preprocessed BOLD in original space*, or just *preprocessed BOLD*. The BOLD time-series were resampled into standard space, generating a *preprocessed BOLD run in [‘MNI152NLin2009cAsym’] space*. First, a reference volume and its skull-stripped version were generated using a custom methodology of *fMRIPrep*. Several confounding time-series were calculated based on the *preprocessed BOLD*: framewise displacement (FD), DVARS and three region-wise global signals. FD and DVARS are calculated for each functional run, both using their implementations in *Nipype* (following the definitions by Power et al. [10]). The three global signals are extracted within the CSF, WM, and whole-brain masks. Additionally, a set of physiological regressors were extracted to allow for component-based noise correction (*CompCor*, [2]). Principal components are estimated after high-pass filtering the *preprocessed BOLD* time-series (using a discrete cosine filter with 128s cut-off) for the anatomical *CompCor* variants: aCompCor. For aCompCor, components are calculated within the intersection of a mask covering the subcortical regions (this subcortical mask is obtained by heavily eroding the brain mask, which ensures it does not include cortical GM regions) and the union of CSF and WM masks calculated in T1w space, after their projection to the native space of each functional run (using the inverse BOLD-to-T1w transformation). Components are also calculated separately within the WM and CSF masks. For each CompCor decomposition, the *k* components with the largest singular values are retained, such that the retained components’ time series are sufficient to explain 50 percent of variance across the nuisance mask (CSF, WM, combined, or temporal). The remaining components are dropped from consideration. The head-motion estimates calculated in the correction step were also placed within the corresponding confounds file. The confound time series derived from head motion estimates and global signals were expanded with the inclusion of temporal derivatives and quadratic terms for each [11]. Frames that exceeded a Euclidian norm of 0.4 mm were annotated as motion outliers. All resamplings can be performed with *a single interpolation step* by composing all the pertinent transformations (i.e. head-motion transform matrices, susceptibility distortion correction when available, and co-registrations to anatomical and output spaces). Gridded (volumetric) resamplings were performed using antsApplyTransforms (ANTs), configured with Lanczos interpolation to minimize the smoothing effects of other kernels [8]. Non-gridded (surface) resamplings were performed using mri_vol2surf (FreeSurfer).

The resulting data were then smoothed with an isometric 6mm Gaussian kernel, scaled to the grand mean intensity and deconvolved. Each block was deconvolved separately. In the deconvolution the following were included as regressors of no interest: a set of basis functions to model signal drift, the motion parameters and their derivatives, Framewise Displacement [9], the first six components from aCompCor (above). In addition, frames (TRs) exceeding a Euclidean norm of 0.4 mm and the immediately preceding frame were excluded from analysis. The regressors of interest were the correct trials of each block, and the coefficient of fit of the correct trials were entered into the group-level analysis.

Response time (RT) and Accuracy: For RT, the only significant effects were Task (F(1,444.2) = 34.41, p<0.0001, η^2^=0.07) and Block (F(5,439.5) = 3.20, p = 0.008, η^2^=0.04). The effect of Task resulted from, as expected, participants responding with longer latencies for the 2-back task (748 ms) than for the 0-back task (587 ms). The effect of Block resulted from shorter latencies on Block1 than on subsequent blocks (mean RT for blocks 1-6: 642, 679, 682, 670, 673, 660 ms). No other effects or interactions were significant.

For accuracy there was a main effect of CF (F(1,319.2) = 5.35, p = 0.02, η^2^=0.02) resulting from a small negative relationship between accuracy and CF (coefficient= -0.01): for each increase in the CF score, participants became 0.01% less accurate. CF also interacted with Age (F(1,348.8) = 4.66, p = 0.03, η^2^=0.01), as shown in Figure S1. In chronologically younger individuals, increasing CF was associated with less accuracy, but this trend was reversed for older individuals. Age also interacted with Task (F(1,443.9) = 5.40, p=0.02, η^2^=0.01), resulting from a negative relationship between accuracy and age for the 2-back task (coefficient= -0.03) and a positive relationship for the 0-back task (coefficient=0.06). That is, the older participants were less accurate in their responses during the 2-back task, but this trend was reversed for the 0-back task.


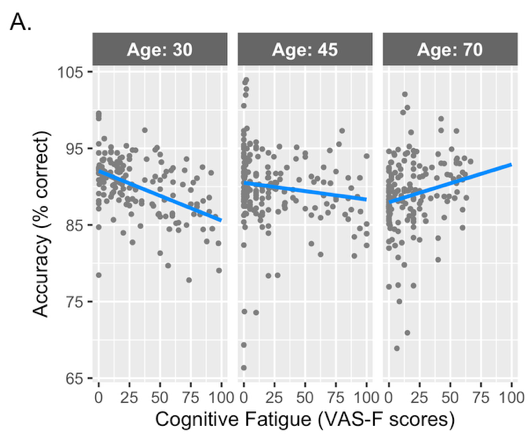


Figure S1. The interaction of CF (VAS-F) and Age in the accuracy data. The continuous variable Age has been divided into tertiles for exposition only (ages 20-39, 40-70 and 71+), with each tertile represented by a column: “Age: 30” shows ages 20-39, “Age: 45” shows ages 40-65, “Age: 70” shows ages 66+. The blue lines show the best fitting regression line for each subset of the data (again, for exposition only).

The only other significant effect was Session (Session: F(1,70.4) = 6.75, p=0.01, η^2^=0.09), which resulted from participants responding with higher accuracy for Session 2 (100%) than Session 1 (94.7%).

Trait CF: There were no significant relationships between scores on the cognitive subscale of the MFIS and age, sex, or site. Additionally, there was no significant relationship between the cognitive subscale of the MFIS and state CF when CF.intercept was added to the model.
